# Assessing the Metabolic Effects of Aromatherapy in Human Volunteers

**DOI:** 10.1155/2013/356381

**Published:** 2013-05-02

**Authors:** Yinan Zhang, Yani Wu, Tianlu Chen, Lei Yao, Jiajian Liu, Xiaolan Pan, Yixue Hu, Aihua Zhao, Guoxiang Xie, Wei Jia

**Affiliations:** ^1^Center for Translational Medicine and Shanghai Key Laboratory of Diabetes Mellitus, Department of Endocrinology and Metabolism, Shanghai Jiao Tong University Affiliated Sixth People's Hospital, Shanghai 200233, China; ^2^School of Agriculture and Biology, Shanghai Jiao Tong University, Shanghai 200240, China; ^3^Center for Translational Biomedical Research, University of North Carolina at Greensboro, Kannapolis, NC 28081, USA

## Abstract

Aromatherapy, a form of complementary and alternative medicine (CAM) that uses essential oils through inhalation, is believed to enhance physical and spiritual conditions. Although clinical studies suggest that the use of essential oils may have therapeutic potential, evidence for the efficacy of aromatherapy in treating medical conditions remains poor, with a particular lack of studies employing rigorous analytical methods that capture its identifiable impact on human biology. Here, we report a comprehensive metabolomics study that reveals metabolic changes in people after exposed to aroma inhalation for 10 continuous days. In this study, the metabolic alterations in urine of 31 females with mild anxiety symptoms exposed to aerial diffusion of aromas were measured by GC-TOF-MS and UPLC-Q-TOF-MS analyses. A significant alteration of metabolic profile in subjects responsive to essential oil was found, which is characterized by the increased levels of arginine, homocysteine, and betaine, as well as decreased levels of alcohols, carbohydrates, and organic acids in urine. Notably, the metabolites from tricarboxylic acid (TCA) cycle and gut microbial metabolism were significantly altered. This study demonstrates that the metabolomics approach can capture the subtle metabolic changes resulting from exposure to essential oils, which may lead to an improved mechanistic understanding of aromatherapy.

## 1. Introduction

Aromatherapy, the practice of using aromatic plant-derived essential oils for a variety of applications including mood enhancement, pain relief, and improved cognitive function, is increasingly being used in complementary and alternative medicine (CAM) units as well as primary care settings [1]. One of the methods used in aromatherapy is release of odor to a particular environment. The proposed mechanism of action of the respiratory administration of aromatherapy begins with the absorption of volatile odor molecules through the nasal mucosa. Odor molecules are then transformed into chemical signals, which travel to the olfactory bulb and then other parts of the limbic system of the brain and the cerebral cortex and the olfactory sensory center at the base of the brain, interacting with the neuropsychological framework to produce characteristic physiological and psychological effects on target tissues [2]. One study investigated the effects of various plant-derived or synthetic odors on task performance, reaction time, and autonomic parameters or evaluated the direct effects of odors on the brain via electroencephalogram patterns and functional imaging studies [3]. A lot of studies have demonstrated the antianxiety effect by essential oils from rose, lavender, lemon, and peppermint [4, 5]. These studies have consistently shown that odors can produce specific effects on human neuropsychological and autonomic function, suggesting that aromatherapy has beneficial effects in the context of stressful and adverse psychological conditions.

In recent years, researchers have studied the components of essential oil that have antianxiety effect and possible molecular principles. Umezu et al. [6] from Japan analyzed the antianxiety components of essential oil from lavender using GC-MS and identified linalool as the main antianxiety pharmacological substance. Komiya et al. [7] studied the antianxiety effect of essential oil from rose, lavender, and lemon. They also researched the linkage of essential oil from lemon with benzodiazepine, 5-hydroxy tryptamine, dopamine, and adrenergic receptor and found essential oil from lemon increasing the nerve-energy of 5-hydroxy tryptamine from the suppression the activity of dopamine. Umezu [8] studied that dopamine might be involved in the mouse ambulation promoted by peppermint oil and its constituents. Wu et al. [9] have done a thorough metabolomic study on rats' brain tissue and urinary responses to aromatherapy. These metabolic changes include the increased carbohydrates and lowered levels of neurotransmitters (tryptophan, serine, glycine, aspartate, histamine, tyrosine, cysteine, phenylalanine, hypotaurine, histidine, and asparagine), amino acids, and fatty acids in brain. Elevated aspartate, carbohydrates (sucrose, maltose, fructose, and glucose), nucleosides (adenine and uridine), and organic acids such as lactate and pyruvate were also observed in urine.

Despite of the numerous results demonstrating beneficial effects on mood and relaxation observed in behavioral and emotional studies, evidence for the efficacy and mechanistic understanding of aromatherapy in treating medical conditions remains poor, with a particular lack of studies employing rigorous methodology [10, 11]. 

Here, we report a metabolomic study designed to evaluate the effect of an essential oil preparation in university female students. Urine samples were collected and analyzed by both gas chromatography time-of-flight mass spectrometry (GC-TOFMS) and ultraperformance liquid chromatography-quadrupole time-of-flight mass spectrometry (UPLC-QTOFMS) in conjunction with multivariate statistical analysis, including principal component analysis (PCA), partial least squares discriminant analysis (PLS-DA), and orthogonal partial least squares-discriminant analysis (OPLS-DA), to obtain significant endogenous metabolite markers of aromas-exposure. 

## 2. Methods

### 2.1. Chemicals and Materials

The essential oil used in the study is the same as used in our previous report [9], prepared from 4 aromatic plants, *Lavandula angustifolia and Salvia sclarea L. *from China, *Santalum album *from India, and *Citrus sinensis *from the United States. The constituents of the essential oil were assayed by gas chromatography (GC) and GC-mass spectrometry (GC-MS) and were mainly limonene, linalool, linalyl acetate, polysantol, and other 30 chemicals (the detailed chemical composition of the essential oil was shown in our previous paper [9]). Acetonitrile and methanol of HPLC grade were obtained from Merck Chemicals (Darmstadt, Germany). Analytical-grade methanol was obtained from the Shanghai Lin Feng Chemical Reagent Co, Ltd. (China). All aqueous solutions were prepared with ultrapure water produced by a Milli-Q system (18.2 MΩ, Millipore, Bedford, MA). Chloroform was analytical grade and purchased from China National Pharmaceutical Group Corporation (Shanghai, China). L-2-Chlorophenylalanine was purchased from Intechem Tech. Co. Ltd. (Shanghai, China). BSTFA (1% TMCS), heptadecanoic acid, and methoxyamine were purchased from Sigma-Aldrich (St. Louis, MO).

### 2.2. Human Sample Treatment

Fifty-two female volunteers who were not allergic to essential oil with an average age of 20.29 ± 1.51 years from Shanghai Jiao Tong University were recruited in this study. They were allowed to do a double blind Symptom Check List-90 (SCL90) test. The checklist consists of nine subscales including somatization (SOM), obsessive-compulsive (O-C), interpersonal sensitivity (I-S), depression (DEP), anxiety (ANX), hostility (HOS), phobic anxiety (PHOB), paranoid ideation (PAR), and psychoticism (PSY). Thirty-one volunteers who have at least one subscale with a score over 2.0 were subject to the further study. We have arranged a quiet classroom (100 m^2^, room temperature = 24 ± 1°C, and room humidity = 45 ± 15%) for the experiment. The doors and windows of the room were closed 15 minutes before the examination and in which the environment was fragranced by incenses of the essential oil. The examination was performed in the atmosphere of the aroma, and the doors and windows of the classroom were kept close until the end of the examination. The duration of the examination was 45 minutes a day for 10 continuously days. All the volunteers were required to eat only blander food in the 10 days and stay quietly (reading and writing) in the room. Urine samples were collected on day 0 (before exposed to essential oil inhalation, BE) and day 10 (after exposed to essential oil inhalation, AE). The protocol was approved by the Shanghai Jiao Tong University Review Board, and all participants gave informed consent before they were involved in the study.

### 2.3. Urine Sample Preparation for GC-TOFMS Analysis

Urine metabolites were chemically derivatized prior to mass spectrometry analysis following our previously published procedure with minor modifications [12]. An aliquot of 100 *μ*L urine sample was spiked with two internal standard solutions (10 *μ*L of L-2-chlorophenylalanine in water, 0.3 mg/mL; 10 *μ*L of heptadecanoic acid in methanol, 1 mg/mL) and vortexed for 10 s. The mixed solution was extracted with 300 *μ*L of methanol/chloroform (3 : 1) and vortexed for 30 s. After storing for 10 min at −20°C, the samples were centrifuged at 12 000 rpm for 10 min. An aliquot of the 300 *μ*L supernatant was transferred to a glass sampling vial to vacuum-dry at room temperature. The residue was derivatized using a two-step procedure. First, 80 *μ*L of methoxyamine (15 mg/mL in pyridine) was added to the vial and kept at 30°C for 90 min, followed by 80 *μ*L of BSTFA (1% TMCS) at 70°C for 60 min.

### 2.4. GC-TOFMS Analysis

A 1 *μ*L aliquot of the derivatized solution was injected in splitless mode into an Agilent 6890 N gas chromatograph coupled with a Pegasus HT time-of-flight mass spectrometer (Leco Corporation, St. Joseph, MI). Separation was achieved on a DB-5ms capillary column (30 m × 250 *μ*m i.d., 0.25 *μ*m film thickness; (5%-phenyl)-methylpolysiloxane bonded and cross-linked; Agilent J&W Scientific, Folsom, CA), with helium as the carrier gas at a constant flow rate of 1.0 mL/min. The temperature of injection, transfer interface, and ion source was set to 270, 260, and 200°C, respectively. The GC temperature programming was set to 2 min isothermal heating at 80°C, followed by 10°C/min oven temperature ramps to 180°C, 5°C/min to 240°C, and 25°C/min to 290°C, and a final 9 min maintenance at 290°C. Electron impact ionization (70 eV) at full scan mode (m/z 30–600) was used, with an acquisition rate of 20 spectra/s in the TOFMS setting.

### 2.5. Urine Sample Preparation for UPLC-QTOFMS Analysis

Urine samples were preliminarily treated following our previously published procedure with minor modifications [12]. The collected urine samples were centrifuged at 13,000 rpm for 10 min at 4°C, and the resulting supernatants were immediately stored at −80°C pending UPLC-QTOFMS analysis. Also, 600 *μ*L of ultrapure water (containing 5 *μ*g/mL L-2-chlorophenylalanine as the internal standard) was added to urine (300 *μ*L) and vortexed for 1 min and then filtered through a syringe filter (0.22 *μ*m) and placed into the sampling vial pending UPLC-QTOFMS analysis. 

### 2.6. UPLC-QTOFMS Analysis

Urine metabolite profiling was performed using a Waters ACQUITY UPLC system equipped with abinary solvent delivery manager and a sample manager (Waters Corporation, Milford, MA), coupled to a Micromass Q-TOF Premiermass spectrometer equipped with an electrospray interface (Waters Corporation, Milford, MA). Chromatographic separations were performed on a 2.1 × 100 mm 1.7 *μ*m ACQUITY BEH C_18_ chromatography column. The column was maintained at 45°C and eluted with a 1%–99% acetonitrile (0.1% (v/v) formic acid)-aqueous formic acid (0.1% (v/v) formic acid) gradient over 10 min at a flow rate of 0.40 mL/min. A 5 *μ*L aliquot sample was injected onto the column. The mass accuracy analysis and detailed MS parameters were optimized according to our previous work [12]. During metabolite profiling experiments, centroid data were acquired for each sample from 50 to 1000 Da with a 0.10 s scan time and a 0.01 s interscan delay over a 10 min analysis time.

### 2.7. Data Analysis

The acquired MS files from GC-TOFMS analysis were exported in NetCDF format by ChromaTOF software (v3.30, Leco Co., CA). CDF files were extracted using custom scripts (revised Matlab toolbox hierarchical multivariate curve resolution (H-MCR), developed by Par Jonsson et al. [13, 14] in the MATLAB 7.0 (The MathWorks, Inc.) for data pretreatment procedures such as baseline correction, denoising, smoothing, alignment, time-window splitting, and multivariate curve resolution (based on multivariate curve resolution algorithm)) [14]. The resulting three-dimensional data set included sample information, peak retention time, and peak intensities. Internal standards and any known artificial peaks, such as peaks caused by noise, column bleed, and BSTFA derivatization procedure, were removed from the data set. Additionally, compound identification was performed by comparing the mass fragments with NIST 05 standard mass spectral databases in NIST MS search 2.0 (NIST, Gaithersburg, MD) software with a similarity of more than 70% and finally verified by available reference compounds.

The UPLC-QTOFMS raw data were analyzed by the MarkerLynx Applications Manager version 4.1 (Waters, Manchester, UK) using parameters reported in our previous work [15]. A list of the ion intensities of each peak detected was generated, using retention time (RT) and the m/z data pairs as the identifier for each ion. The resulting three-dimensional matrix contained arbitrarily assigned peak indexes (retention time-m/z pairs), sample names (observations), and ion intensity information (variables). To obtain consistent differential variables, the resulting matrix was further reduced by removing any peaks with missing value (ion intensity) in more than 40% of the samples from both groups. The ion peaks generated by the internal standard were also removed. The data was then normalized by the sum of all peak intensities within the sample.

Then, the GC-TOFMS and UPLC-QTOFMS were put together and unit variance scaled during chemometric data analysis in the SIMCA-P + 13.0 Software package (Umetrics, Umeå, Sweden). Partial least squares-discriminant analysis (PLS-DA) was carried out to discriminate between different groups. On the basis of a variable importance in the projection (VIP) threshold of 1 from the PLS-DA model, a number of metabolites responsible for the difference in the metabolic profiles between two groups could be obtained. In parallel, the metabolites identified by the PLS-DA model were validated at a univariate level using the Student's *t*-test with the critical *P*  value set to 0.05. The resultant *P*  values for all metabolites were subsequently adjusted to account for multiple testing. The corresponding fold change shows how these selected differential metabolites varied between groups. 

## 3. Results and Discussion 

### 3.1. SCL-90 Test

The 31 volunteers were given the test before being exposed to essential oil inhalation and after being exposed to essential oil inhalation for 10 days, and the result was shown in Table 1. The values of normal Chinese University students were also included [16]. The BE values of the 31 volunteers are a little bit higher than the normal values which indicated that they were under slight anxiety. The AE values are lower or similar to the normal value which indicated they relieved their stress after inhaling the essential oil for 10 days.

### 3.2. Metabolic Profile of GC-TOFMS and UPLC-QTOFMS Analysis

**Figure 1:**
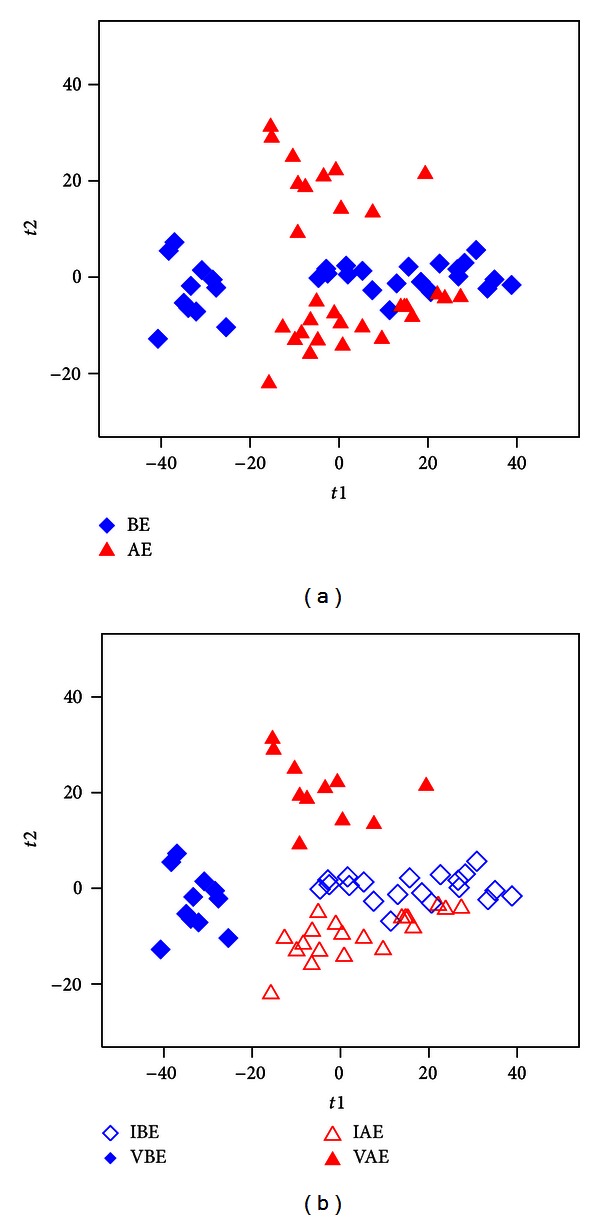
Metabolic profiles depicted by PLS-DA scores plot of GC-TOFMS and UPLC-QTOFMS spectral data from human urine of (a) BE group and AE group; (b) IBE group, VBE group, IAE group, and VAE group.

A wide range of carbohydrates, amino acids, organic acids, and alcohols were detected using GC-TOFMS and UPLC-QTOFMS analyses of urine. Among a total of 388 chromatographic features obtained from the GC-TOFMS spectra of urine samples, 134 metabolites were identified with NIST 05 standard mass spectral databases with a similarity >70%, and 83 were further verified by available reference standards, respectively. Altogether, 5690 peaks were detected with our optimized UPLC-QTOFMS analysis protocol. And we were able to confirm 111 from reference standards and HMDB. These two datasets were normalized and put together, and at last 215 metabolites were identified after combination.  Figure 1(a) illustrates the scores plots of PLS-DA model of the subjects from BE and AE group. It is showed that 11 people are vulnerable to essential oil (Vulnerable group before being exposed to essential oil inhalation, VBE, and Vulnerable group after being exposed to essential oil inhalation, VAE), while the other 20 are invulnerable to essential oil (Invulnerable group before being exposed to essential oil inhalation, IBE, and Invulnerable group after being exposed to essential oil inhalation, IAE) (Figure 1(b)). 

**Figure 2:**
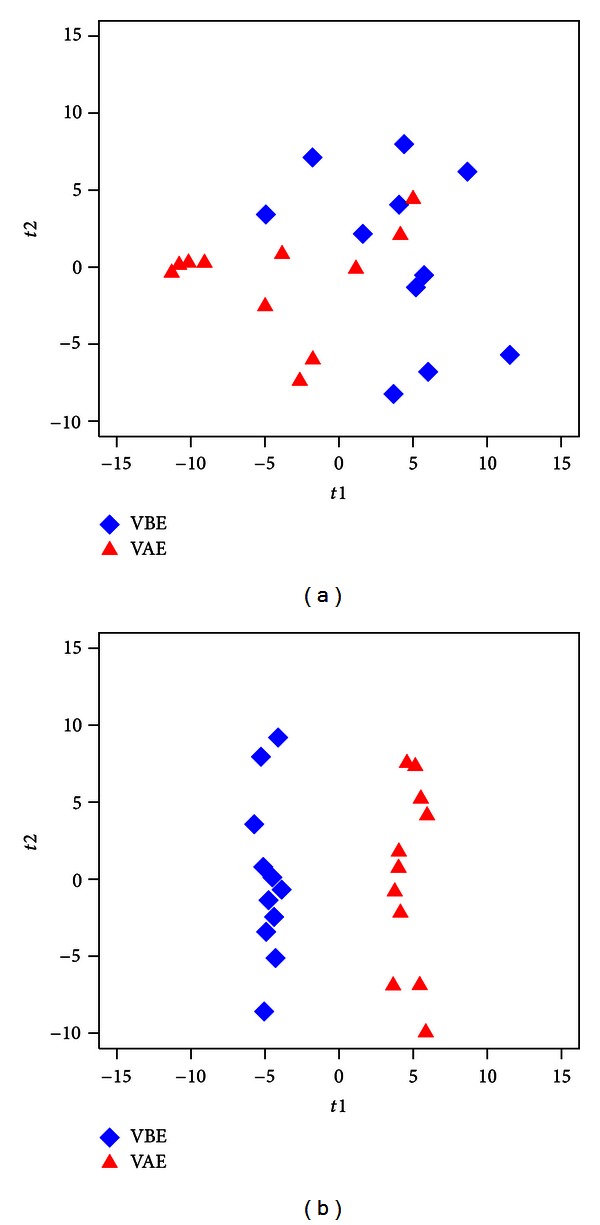
Metabolic profiles depicted by PCA scores plot (a) and OPLS scores plot (b) from the VBE group and VAE group with only identified metabolites.

The trend of separation can be seen from the PCA scores plot between VBE and VAE groups using only identified metabolites as shown in Figure 2(a) (R2X = 0.306). We selected the differentially expressed urine metabolites in the VBE group relative to VAE group based on the VIP values (VIP > 1) by OPLS models with 1 predictive component and 2 orthogonal components (R2X = 0.333, R2Y = 0.979, and Q^2^(cum) = 0.484) (Figure 2(b)) constructed with the identified metabolites. Univariate statistical analysis, Student's *t*-test, was performed on these metabolites to evaluate their significance. Differentially expressed metabolites in urine were obtained with a *P*  value less than 0.05 (Table 2). All these metabolites remained statistically significant after multiple testing. On the other hand, there is no separation trend between IBE and IAE groups from the PCA scores plot, and the Q^2^(cum) of the OPLS model is below zero; these illustrated that there is no significant difference between IBE and IAE groups on the level of metabolites.

### 3.3. Daily Exposure to Aromas Induces Significant Metabolic Changes

**Figure 3:**
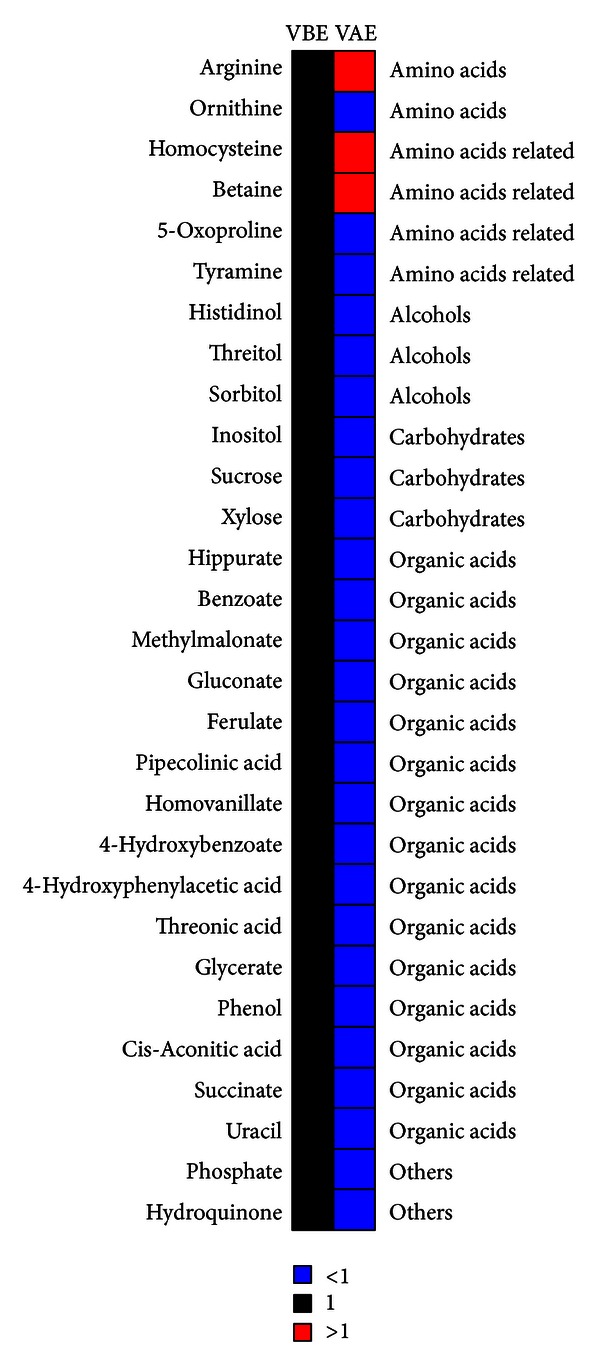
Heat map showing changes in metabolites in urine after being exposed to essential oil inhalation for 10 days (VAE group) to themselves before being exposed to essential oil inhalation (VBE group). Shades of red and blue represent fold increase and fold decrease of a metabolite, respectively, in VAE group relative to VBE group (see color scale).

We detected 29 differentially expressed metabolites induced by aromas inhalation in human urine (Figure 3 and Table 2), which include a number of carbohydrates, alcohols, organic acids, and amino acids. As compared to themselves before being exposed to essential oil inhalation, people after being exposed to essential oil inhalation were characterized by higher levels of arginine, homocysteine, and betaine, lower levels of alcohols (threitol, sorbitol, and histidinol), carbohydrate (inositol, sucrose, and xylose), pyrimidine (uracil), and organic acids (hippurate, benzoate, methylmalonate, gluconate, ferulate, pipecolinic acid, homovanillate, 4-hydroxybenzoate, 4-hydroxyphenylacetic acid, threonic acid, glycerate, phenol, cis-aconite acid, and succinate).

Aromatherapy, a form of inexpensive and noninvasive CAM, uses of essential oils, the scented, volatile liquid substances extracted from plants using steam or pressure, which dates back centuries for the purpose of altering a person's mind, mood, cognitive function, or health [17]. Although there were already some findings on the pharmacological effect and mechanism of essential oil, most of them were in the field of behavioral and emotional science [18–20]. The effects of essential oil were evaluated from senses and experiences; thus, it is hard to scientifically assess and explain aromatherapy. This paper, however, is focused on using recent developed metabolomics technology to evaluate this CAM intervention by understanding metabolic variations. 

From our previous publication [11], we found that alkenes, esters, and alcohols are the main constituents of the essential oil. None of them has been detected in our profiling data in human urine, which means that all the changes that we found were due to endogenous change and not due to breathe in extrinsic substances.

The result of SCL90 (Table 1) demonstrated that ANX and PHOB from 31 people after being exposed to essential oil inhalation were significantly different from themselves before being exposed to essential oil inhalation. Also, O-C and DEP were significantly different as well. This indicated that the essential oil used in this study not only relaxes anxious moods, but also has some effect on other psychological health. It does have some effect on mood adjustment, and it is in agreement with previous publications [4, 19, 20].

However, from our metabolomics results (Figure 1), it is found that some people's metabolic profiles were not changed much after aromatherapy while others were significantly altered. This is because individual experience of an odour may also affect response. The marked association of odours with emotional response is due to the prominence of afferent links from the olfactory bulb to the amygdala, where emotional significance is attached to incoming stimuli [21]. Consequently, study participants for whom a particular odour has strong negative (or positive) associations may be expected to introduce further interindividual variability in outcome measures [22]. Hence, only eleven people out of 31 were susceptible to aromatherapy of this specific essential oil (Figure 1(b)). 

An interesting finding of our analysis suggests the decreased carbohydrates in urine that are critically involved in the metabolic perturbation are derived from inhalation of aromas. Carbohydrates have been shown in several reports that have anxiolytic effects [23], and the significantly decreased levels of carbohydrates in urine may be due to the therapeutic effects of aromatherapy on anxiety. VAE group has a much lower level of Inositol than the VBE group. Myoinositol has been shown to have antidepressant and anxiolytic activities in both humans and animals [24, 25]. Also of interest is the finding that several energy metabolism related metabolites, including cis-aconitic acid, succinate, and hydroquinone, were found at different levels between the VAE and VBE groups. It was found that oxidative stress was involved in the pathogenesis of neurological diseases, such as psychiatric disorders [26, 27] and anxiety [28, 29]. Oxidative stress is caused by altered mitochondrial energy pathways leading to abundant reactive oxidative stress compounds. It is therefore not surprising that metabolites and carbohydrates involved in TCA cycle were found to be significantly different between the VAE and VBE groups.

It is believed that there was a strong relationship between gastrointestinal symptoms and anxiety [30]. Changes of gastrointestinal functional ecology are directly linked to gut microbial populations and activities [31]. Urine of mammals contains many polar metabolites resulting from gut microbial-mammalian cometabolism [31, 32]. Therefore, metabolic variations of urinary excretion of many aromatic compounds (e.g., phenolics, indoles, and benzoyl derivatives) provide indirect information on the gut microbial metabolic activities [33]. Hippurate, 4-hydroxybenzoate, 4-hydroxyphenylacetic acid, benzoate, tyramine, and phenol were all significantly decreased in VAE group, reflecting an altered gut microbial metabolism associated with aromatherapy. Metabolite profiling of urine in this study revealed an entire lower level of gut microbe related metabolites after being exposed to essential oil inhalation for 10 days. 

The aim of this study was to obtain metabolite markers in urine of human exposed to essential oil to gain mechanistic insights into the metabolic impact of aromatherapy. However, there are limitations in the current metabolomic study. First, the sample size was a little bit small especially for people vulnerable to this essential oil. More people and longer essential oil inhale time may be tested in future study. Second, only urine was used in the experiments, and because of the blood-brain barrier, the metabolism of the brain is independent of peripheral circulation, and, hence, the metabolic variances induced by aromas inhalation were different between urine and brain. Additional mechanistic information and metabolite markers may be identified with brain tissue. 

 In conclusion, we identified the global metabolic responses to aromatherapy characterized by unique metabolic signatures in human urine involving carbohydrates, organic acids, amino acids, and pyrimidine. The metabolites involved in TCA cycle and gut microbe metabolism were significantly decreased after being exposed to essential oil inhalation for 10 days. These distinctions collectively constitute a metabolic window into essential oil effect, providing metabolic endpoints that complement the interpretation of behavioral research. The results of this study also highlight the potential of this sufficiently robust and noninvasive profiling approach for research on the CAM of aromatherapy.

## Figures and Tables

**Table 1:** SCL-90 test checklist for volunteers before being exposed to essential oil inhalation and after being exposed to essential oil inhalation for 10 days.

Subscales	BE	AE	*P*	Norm of China (2008) (*n* = 9941)
SOM	1.59 ± 0.40	1.37 ± 0.36	0.03*	1.45 ± 0.49
O-C	2.40 ± 0.63	1.93 ± 0.66	0.01*	1.98 ± 0.63
I-S	2.80 ± 0.61	1.75 ± 0.63	0.04*	1.88 ± 0.63
DEP	1.97 ± 0.61	1.68 ± 0.54	0.05	1.74 ± 0.62
ANX	2.03 ± 0.73	1.65 ± 0.65	0.03*	1.61 ± 0.55
HOS	1.83 ± 0.66	1.49 ± 0.45	0.02*	1.61 ± 0.62
PHOB	1.55 ± 0.55	1.29 ± 0.39	0.03*	1.38 ± 0.49
PAR	1.74 ± 0.58	1.58 ± 0.48	0.22	1.72 ± 0.62
PSY	1.86 ± 0.60	1.57 ± 0.45	0.04*	1.59 ± 0.54
OTHER	1.98 ± 0.70	1.66 ± 0.59	0.06	—

Compared with BE; *P < 0.05.

**Table 2:** List of differential metabolites in urine after being exposed to essential oil inhalation for 10 days (VAE group) to themselves before being exposed to essential oil inhalation (VBE group).

Classes	Metabolites	VIP^1^	FC^2^	*P* ^ 3^
Amino acids	Arginine	1.80	3.76	2.44*E* − 02
	Ornithine	1.78	0.50	2.66*E* − 02

Amino acids related	Homocysteine	1.98	2.05	1.21*E* − 02
	Betaine	2.21	1.79	4.21*E* − 03
	5-Oxoproline	2.03	0.61	9.76*E* − 03
	Tyramine	2.58	0.47	4.49*E* − 04

Alcohols	Histidinol	1.66	0.34	4.02*E* − 02
	Threitol	2.77	0.48	9.66*E* − 05
	Sorbitol	1.98	0.16	1.19*E* − 02

Carbohydrates	Inositol	2.48	0.53	8.67*E* − 04
	Sucrose	1.97	0.27	1.29*E* − 02
	Xylose	1.90	0.61	1.68*E* − 02

Organic acids	Hippurate	1.99	0.17	1.14*E* − 02
	Benzoate	2.28	0.34	2.90*E* − 03
	Methylmalonate	1.94	0.62	1.42*E* − 02
	Gluconate	1.76	0.70	2.86*E* − 02
	Ferulate	1.65	0.37	4.20*E* − 02
	Pipecolinic acid	1.62	0.74	4.60*E* − 02
	Homovanillate	2.34	0.40	2.11*E* − 03
	4-Hydroxybenzoate	1.75	0.48	2.93*E* − 02
	4-Hydroxyphenylacetic acid	1.99	0.44	1.14*E* − 02
	Threonic acid	2.52	0.57	6.78*E* − 04
	Glycerate	2.41	0.56	1.38*E* − 03
	Phenol	1.80	0.49	2.42*E* − 02
	Cis-Aconitic acid	1.91	0.53	1.65*E* − 02
	Succinate	2.16	0.52	5.23*E* − 03

Pyrimidine	Uracil	1.99	0.54	1.16*E* − 02

Others	Phosphate	2.24	0.69	3.48*E* − 03
	Hydroquinone	1.74	0.55	3.08*E* − 02

^1^Variable importance in the projection (VIP) was obtained from PLS-DA model with a threshold of 1.0;

^2^fold change (FC) was obtained by comparing those metabolites in VAE group to VBE group;

^3^ *P* values were calculated from Student's *t*-test;

FC with a value >1 indicates a relatively higher concentration present in VAE group, while a value <1 means a relatively lower concentration as compared to VBE.

## Abbreviations

GC-TOF-MS: Gas chromatography time-of-flight mass spectrometry
UPLC-Q-TOFMS: Ultraperformance liquid chromatography-quadrupole time-of-flight mass spectrometry
CAM: Complementary and alternative medicine
PLS-DA: Partial least squares discriminant analysis
PCA: Principal component analysis
OPLS-DA: Orthogonal partial least squares-discriminant analysis
BE: Before exposed to essential oil inhalation
AE: After exposed to essential oil inhalation
VBE: Vulnerable group before exposed to essential oil inhalation
VAE: Vulnerable group after exposed to essential oil inhalation
IBE: Invulnerable group before exposed to essential oil inhalation
IAE: Invulnerable group after exposed to essential oil inhalation.
